# P-2231. The Impact of Cell-free DNA Sequencing in the Evaluation and Management of Infections

**DOI:** 10.1093/ofid/ofae631.2385

**Published:** 2025-01-29

**Authors:** Maurice Marshall, Lea M Monday, Matthew Laporte, Marilynn Fairfax, Timothy R Kennedy, Nahed M Abdel-Haq, Hossein Salimnia, Sorabh Dhar

**Affiliations:** Detroit Medical Center/Wayne State University, West Bloomfield, Michigan; Wayne state University School of Medicine, Detroit, Michigan; Detroit Medical Center/Wayne State University, West Bloomfield, Michigan; Detroit Medical Center/Wayne State University, West Bloomfield, Michigan; Wayne State University Pathology, Detroit, Michigan; Children's Hospital of Michigan/Wayne State University, Detroit, MI; Detroit Medical Center/Wayne State University, West Bloomfield, Michigan; Wayne State University/Detroit Medical Center, John Dingell VAMC, Detroit, Michigan

## Abstract

**Background:**

Cell-free DNA Sequencing is a novel diagnostic test that utilizes next generation sequencing to detect pathogens directly from plasma. Microbial cell-free DNA (mcfDNA) can be detected for a large number of pathogens with high analytic sensitivity and specificity; however, testing may be cost prohibitive and the direct impact on clinical care decisions is unclear.

**Figure:**
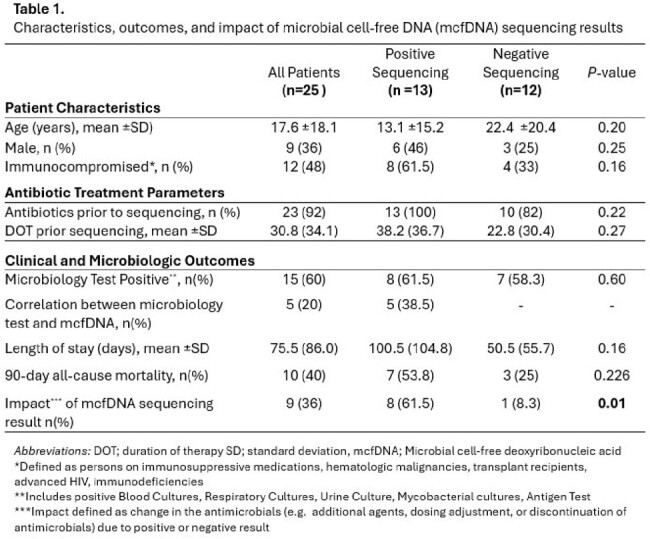

**Methods:**

A retrospective review was performed at a large inner-city tertiary care medical center from 9/2021 to 3/2024 to assess the results of mcfDNA sequencing (using the Karius Test®) on committee approved patients for whom infection was suspected, and without a definitive diagnosis on traditional microbiologic testing or in patients not improving on current antimicrobial therapy. Clinical, microbiologic, and outcome data were collected, and the impact of a positive or negative test on clinical care decisions was abstracted. Test results were defined as impactful if they led to change in the antimicrobials (e.g. additional agents, dosing adjustment, or discontinuation of antimicrobials).

**Figure:**
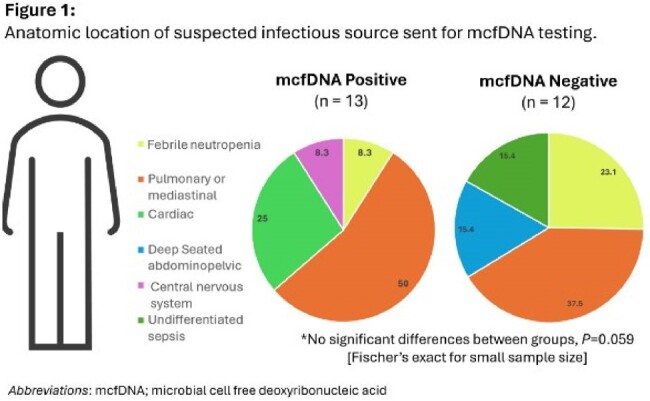

**Results:**

Testing was completed on 25 patients, ranging from 5 months to 63 years (mean age 17 years), 16 (64%) female, 12 (48%) immunocompromised. The average result reporting time was 2.6 days. 23 (92%) patients received antibiotics prior to testing with an average duration of 30.8 days. A total of 13 (52%) of tests were positive by mcfDNA (with a mean of 1.6 organisms (SD=0.65) detected), and 7 (53.8%) polymicrobial results. The positive vs negative did not vary by suspected site, though sample size was small (Fig1). A total of 15 (60%) patients were positive by traditional microbiology tests with an organism correlation of 5 (20%). The mcfDNA results were impactful in 9 (36%) of the patients. A positive test was more likely to be impactful than a negative test (p=0.01). Ninety-day mortality was noted in 10 (40%) of patients and the average duration of hospitalization was 75.5 days – with no difference among patients with a positive mcfDNA (Table 1).

**Conclusion:**

A positive mcfDNA sequencing result was noted to be clinically impactful in 36% of cases. Our data suggests that testing would be most beneficial for patients that lack a definitive infectious diagnosis or those not responding to appropriate therapy.

**Disclosures:**

All Authors: No reported disclosures

